# Calomel in Sickness of Pregnancy

**Published:** 1860-09

**Authors:** 


### Calomel in Sickness of Pregnancy.

The worst form of this very troublesome sickness was relieved entirely by Dr. C. A. Bagot, (*Dublin Med. Press*,) with Calomel, pushed to slight salivation.

